# Interventional studies for preventing surgical site infections in sub-Saharan Africa – A systematic review

**DOI:** 10.1016/j.ijsu.2012.04.004

**Published:** 2012

**Authors:** Alexander M. Aiken, David M. Karuri, Anthony K. Wanyoro, Jana Macleod

**Affiliations:** a London School of Hygiene and Tropical Medicine, Keppel Street, London, UK; b Kenya Medical Research Institute – Wellcome Trust Research Programme, Kenya; c Muriranja District Hospital, Murang’a, Kenya; d Aga Khan University College of Health Sciences, Kenya; e Kenyatta University, Kenya; f Thika Level 5 Hospital, Thika, Kenya

**Keywords:** Surgical site infections, Sub-Saharan Africa, Interventional studies, Systematic review

## Abstract

**Background:**

There is a great need for safe surgical services in sub-Saharan Africa, but a major difficulty of performing surgery in this region is the high risk of post-operative surgical site infection (SSI).

**Methods:**

We aimed to systematically review which interventions had been tested in sub-Saharan Africa to reduce the risk of SSI and to synthesize their findings. We searched Medline, Embase and Global Health databases for studies published between 1995 and 2010 without language restrictions and extracted data from full-text articles.

**Findings:**

We identified 24 relevant articles originating from nine countries in sub-Saharan Africa. The methodological quality of these publications was diverse, with inconsistency in definitions used for SSI, period and method of post-operative follow-up and classification of wound contamination. Although it was difficult to synthesise information between studies, there was consistent evidence that use of single-dose pre-operative antibiotic prophylaxis could reduce, sometimes dramatically, the risk of SSI. Several studies indicated that alcohol-based handrubs could provide a low-cost alternative to traditional surgical hand-washing methods. Other studies investigated the use of drains and variants of surgical technique. There were no African studies found relating to several other promising SSI prevention strategies, including use of checklists and SSI surveillance.

**Conclusions:**

There is extremely limited research from sub-Saharan Africa on interventions to curb the occurrence of SSI. Although some of the existing studies are weak, several high-quality studies have been published in recent years. Standard methodological approaches to this subject are needed.

## 1 Introduction

Performing surgery in sub-Saharan Africa has many challenges different from those encountered in high-income countries: costs are usually severely constrained; numbers of trained theatre staff are generally low and facilities are often rudimentary. However, one of the principal difficulties for the surgeon in sub-Saharan Africa is the high risk of post-operative surgical site infection (SSI). In two recent WHO-led review papers, the risk of SSI in developing countries was *“strikingly higher than in equivalent surgical procedures in high-income countries”*^1^ and the problem was found to be particularly acute in sub-Saharan Africa.^2^ Although extensive research into SSI prevention has been conducted in high-income countries, we were aware of few interventional studies that had been conducted in sub-Saharan Africa. As SSI constitutes a major challenge for surgeons in African countries, we felt this might represent a significant “knowledge gap” in clinical science.

We therefore set out to summarise interventional studies conducted in sub-Saharan Africa that had attempted to reduce the risk of SSI. We systematically reviewed publications relating to this topic to collate the existing African research for the general surgical audience, and also outline the way forward for future studies addressing this important issue.

## 2 Methods

### 2.1 Search strategy

**Fig. 1:**
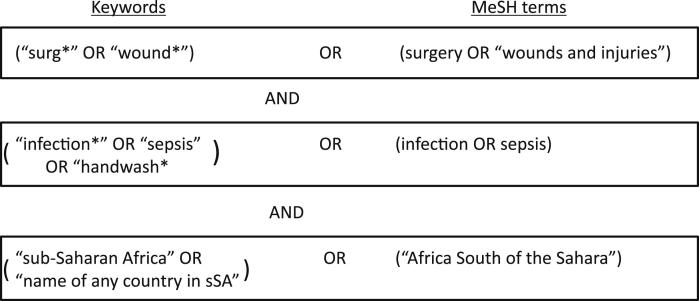
Search items used for systematic review.

We aimed to identify all recent publications giving information on interventions used to reduce the risk of SSI where the research was conducted in countries in sub-Saharan Africa (sSA), without restriction to type of surgery or intervention. We searched Medline, Embase and Global Health databases for reports published between January 1995 and December 2010 with no language restrictions. We used search terms as shown in Fig. 1.

Each title/abstract was screened by two of the authors and a decision on which full-text articles to retrieve was reached after discussion amongst all authors. Additional searches were performed using the reference sections of identified publications and the authors’ own knowledge of the area. Articles were defined as “interventional” studies if the full-text manuscript contained information on at least two groups of patients for whom different management (of whatever type) had been used in an attempt to reduce the risk of SSI after any type of surgical procedure. “Interventional studies” were not limited to randomised controlled trials (RCT) – other direct comparisons (e.g. “before and after” studies) were also included.

### 2.2 Inclusion + exclusion criteria

We included interventional studies conducted in sSA, published between 1995 and 2010. We excluded studies where occurrence of SSI was not a major focus of the intervention. We excluded multicentre studies where data from African sites was not presented separately. We did not exclude any surgical specialities or reject any articles based on quality criteria.

## 3 Results

### 3.1 Search findings

**Fig. 2:**
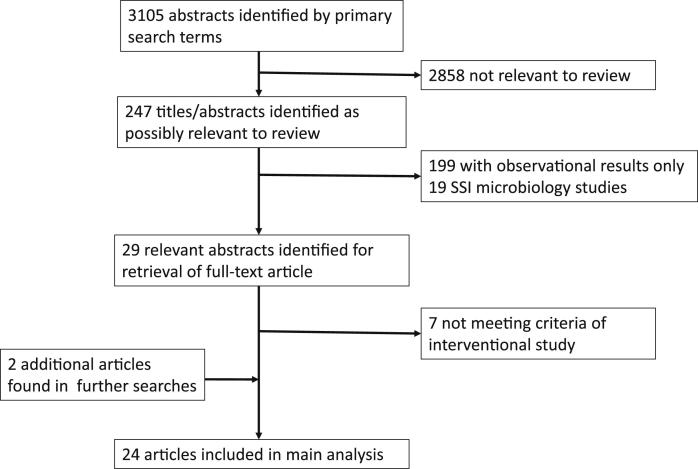
Flow diagram for selection of articles.

Our search yielded 3105 abstracts, of which 247 were judged to be of possible relevance. From these 247, further abstracts were excluded as they contained purely descriptive data (i.e. no comparison of treatments/managements; *n* = 199) or microbiological reports of SSI in sSA (*n* = 19) (Fig. 2). Full-text articles were retrieved for 29 studies, of which seven made use of external comparison groups and therefore did not meet our definition of “interventional”. Two further studies were identified from additional searches. A total of 24 studies in English and French were included.

Studies originated from nine different countries, most frequently Nigeria (*n* = 8), followed by South Africa (*n* = 5), Côte d’Ivoire (*n* = 2), Kenya (*n* = 2), Tanzania (*n* = 2) and Uganda (*n* = 2). There was one study included from each of Ethiopia, Ghana and Mozambique. Studies were written in English (*n* = 22) and French (*n* = 2). For ease of presentation, we separated the studies identified into those relating to the use of antibiotic prophylaxis (*n* = 10), pre-operative interventions (*n* = 4), intra-operative interventions, including different surgical techniques and devices (*n* = 6) and post-operative interventions (*n* = 4) – see Tables 1–4, respectively, arranged by year of publication.

### 3.2 Comparison of studies: methodology

#### 3.2.1 Study design

Patients undergoing a variety of surgical procedures formed the subjects for these studies: the majority of studies examined the effects of an intervention in a single type of surgery, most frequently Caesarean sections (*n* = 11). Most studies were conducted in a single centre and used an individually randomised, controlled trial (RCT) design. One study used cluster randomisation (by operating theatre), two studies used a “before and after intervention” design and one study allowed surgeons to select their operating technique (relating to peritoneal closure) and passively observed results. Two studies of antibiotic prophylaxis used a placebo-control group, whilst all other studies used a recognised standard treatment as the control or baseline arm.

#### 3.2.2 RCT components

There were marked variations in the key elements of RCT design and execution. Some studies clearly described the efforts made to achieve single blinding (investigator only) or double blinding (investigator and participant), although for some operative procedures, it would clearly be impossible to blind the surgeon to the treatment status. A well-conducted RCT of an antibiotic prophylaxis intervention in South Africa achieved double blinding by using a placebo solution with the same appearance as the antibiotic agent.^3^ The actual method used for randomisation was reported in 77% of RCTs (17/21), although two RCTs randomised by allocating alternate patients to the intervention and control arms (alternating assignment).^4,5^ No RCTs were designed from the outset as therapeutic equivalence studies, but several studies finding no significant difference between intervention and standard treatments were interpreted by the authors as providing evidence of equivalence. Only one study^6^ reported adherence to the CONSORT guidelines, which were first published in 1996.^7^

#### 3.2.3 Study size

The total number of patients included ranged from 50 to 3317 subjects, and most studies (17/24, 71%) did not include a sample size calculation.

#### 3.2.4 SSI definitions

There was no consistent usage of any standard schema for defining or classifying surgical site infections. Eight studies provided their own definitions of what they judged to be an SSI, seven studies made reference to a schema described elsewhere and nine studies did not provide any (clear) definition of what they considered as an SSI. The most commonly referenced external schema for SSI classification was that of the Centres for Disease Control (CDC)^5^ – this was referred to by 3 studies.

#### 3.2.5 Follow-up

The post-operative follow-up period and the methods employed to achieve this were also highly variable. Follow-up periods used ranged from 5 days to 12 months. In most studies the follow-up period included both inpatient and outpatient periods (15/24, 63%), though the intensity of effort in outpatient follow-up was diverse. Five studies only followed up patients until discharge and five studies did not report how follow-up was performed. In order to achieve high levels of post-discharge follow-up, one study in Côte d’Ivoire reviewed patients on alternate days up to 30 days after their operation ^8^ and a study in Tanzania provided the transport fare and a free meal for participants who attended their 30-day post-operative review.^9^ A study in Kenya used telephone calls to contact patients after discharge,^6^ though no information was provided on the sensitivity or specificity of this method with respect to a gold standard of “in-person” physician or nurse review.

#### 3.2.6 Wound contamination

Few studies (*n* = 4) made use of a schema for stratifying patients by degree of wound contamination, such as the Surgical Wound Class,^10^ though some studies stated that they excluded patients with unusually contaminated surgical wounds.

### 3.3 Comparison of studies: effects of interventions

It is challenging to summarise the effects of these different interventions due to the variation in SSI definitions, follow-up periods and methods between studies and the failure of most studies to describe the extent of wound contamination.

#### 3.3.1 Antibiotic prophylaxis (Table 1)

The most commonly examined intervention for preventing SSI was the use of antibiotic prophylaxis (*n* = 10), either in comparison to a placebo treatment, or more normally in comparison to an alternative prophylaxis regime. Many different drug regimes were examined, in the context of a variety of different surgical procedures. Precise information about actual timing of dose administration and re-dosing during long procedures was only given in 2/10 studies. Several studies compared the use of a single-dose pre-operative intravenous administration regime against a “standard” regime of prolonged post-operative antibiotic prophylaxis. All of these studies found that a single-dose pre-operative dosing regime was superior to a prolonged post-operative regime, either in terms of reduced use of drugs^9,11–15^ or reduced risk of SSI.^9,16^ One study in Tanzania showed a pronounced effect of implementation of a single-dose pre-operative amoxillin/clavulanate prophylaxis regime: the risk of SSI declined from 21.6% to 4%.^9^ Studies where the use of post-operative antibiotic prophylaxis was avoided or restricted reported no adverse effects of such a restriction. Both the placebo-controlled trials, including one study of cefoxitin prophylaxis for C-section patients in South Africa,^3^ found no benefit of use of antibiotic prophylaxis over placebo.

#### 3.3.2 Pre-operative interventions (Table 2)

Amongst the (non-antibiotic) pre-operative interventions (*n* = 4), two studies on the use of an alcohol-based handrub as an alternative to the traditional surgical hand-washing agents gave consistent results. A large cluster-randomised trial conducted in Kenya^6^ showed no significant difference in the risk of SSI when an alcohol-based handrub was substituted for the traditional soap + water used for the pre-operative surgeon’s hand-wash. Costs for the alcohol handrub were found to be similar to traditional hand-washing method, and the authors noted that alcohol handrub might be much more convenient for institutions where water supply was erratic. A “before and after” study in Côte d’Ivoire^8^ provided similar results and judged that alcohol handrub would be much more cost-effective for an institution to provide. A study in Nigeria^17^ examined the use of a locally produced soap + methylated spirit preparation for use in cleaning the patient’s skin pre-operatively, in comparison to (much more expensive) povidone-iodine – no difference in the risk of SSI was detected. No studies examined the use of pre-operative checklists as a tool for making surgery safer.

#### 3.3.3 Intra-operative interventions (Table 3)

Studies relating to intra-operative interventions (*n* = 6) mainly related to different operative techniques. Four studies^5,18–20^ examined two alternative surgical techniques that might reduce the operating time in Caesarean sections (peritoneal non-closure and the Misgav-Ladach incision) – all studies reported shorter duration of surgery without elevated SSI risk in the experimental arm of the study with elevated SSI risk. One study in Uganda^21^ compared the use of two different ventriculo-peritoneal (VP) shunts – one system was almost 20 times cheaper than the other with an equivalent risk of shunt complications including blockage, device infection and SSI. One study in South Africa^22^ described an experimental technique for circumcision of adults (Tara-Klamp) – this was found to have many drawbacks, including higher risk of SSI.

#### 3.3.4 Post-operative interventions (Table 4)

Amongst studies examining post-operative interventions to reduce the risk of SSI (*n* = 4), three studies examined the use of wound drains in the post-operative period^23–25^ – we considered these as a “post-operative” intervention, although drains were inserted intra-operatively. None of these studies found a benefit in terms of reduced risk of post-operative complication with more extensive use of drains, and one study found higher risk of SSI in patients with drains (for thyroid surgery). There appears to be consistent evidence that the use of post-operative wound drains should be as conservative as possible in an African surgical setting. No studies on post-operative SSI surveillance as a method of reducing SSI risk at the institutional level were found.

## 4 Discussion

Over a 15-year review period, we found only 24 studies describing interventional studies conducted in sub-Saharan Africa for reducing the risk of post-operative SSI, although ten of these were from the last three years of the review period (2008–2010).

### 4.1 Limitations of studies

There were many common errors in designing interventional research studies relating to SSI in Africa. For example, two studies used alternating assignment to “randomise” patients – this is not a suitable method as it allows easy prediction of which treatment the patient will receive. Many studies were likely to be too small to properly evaluate the effect of their intervention on the primary outcome (under-powered) – this could have addressed by performing proper sample size calculations or by combining studies across several sites. However, larger trials are more expensive and multicentre studies present their own logistical challenges. A common misunderstanding in trial interpretation was that failure to find a difference does not mean proof of equivalence – special trial designs (non-inferiority or equivalence trials) are needed to prove equivalence.

The lack of consistency of SSI definitions, follow-up methods and time-periods makes comparisons between these existing studies difficult. Few studies used comparable definitions of what was considered as an SSI and how these were detected. The degree of contamination of the surgical wound is known to be an extremely strong predictor of the risk of SSI in low-income settings,^1^ so use of such a standard stratification system would have facilitated comparisons of the effect of interventions.

### 4.2 Potential future improvements

Some solutions to these problems that could be applied in the future are as follows: adoption of the standard definitions and classification of SSI as provided by the CDC^26^ and of the Surgical Wound Class as used in various studies.^10^ The CDC defines SSI as an infection at the site of the operation within 30 days of the procedure or within 12 months if there is implanted material – universal adherence to this follow-up period would facilitate comparison between studies. In low-resource settings in sSA, it may be difficult to achieve post-operative follow-up when travelling to clinic appointments is prohibitively expensive for patients. Some innovative approaches to post-discharge follow-up (such as contacting patients by telephone) identified in this review may be suitable for further examination in an African context – these need further examination of their sensitivity and specificity in detecting SSI in this context.

### 4.3 Research findings

The existing African research on SSI prevention does provide some important messages which need wide dissemination. Correct use of surgical antibiotic prophylaxis (i.e. single dose, pre-operative delivery) can, in some circumstances, lead to very dramatic reductions in the risk of SSI and can also reduce costs for the patient or institution. This goes directly against the widely held belief amongst African surgeons [in our experience] that “poor hygiene” or crowding in their wards necessitates prolonged post-operative antibiotic usage. Two studies showing no benefit of pre-operative antibiotic prophylaxis over placebo serve to remind prophylaxis regimes are not universally efficacious – locally appropriate agents must be determined. Improved use of antibiotic prophylaxis across sub-Saharan Africa could cut the risk of SSI and simultaneously conserve precious (antibiotic) resources. Use of alcohol handrubs has been shown in two studies to be equivalent (in terms of SSI risk) to traditional soap + water for pre-operative hand-washing by the surgeon and may lead to cost-savings for the institution – this low-cost technology deserves further evaluation across the continent. Use of post-operative drains should be sparing and early discharge should be encouraged, where possible. Some variations in surgical technique were found to be promising, but need more extensive evaluation of their acceptability to surgeons and patients. Some “low-cost” alternative surgical implants and consumables appeared to be equivalent to the standard versions.

Many of these findings are consistent with research and guidelines for preventing SSI originating from high-income settings.^27–31^ No research studies examining the use of checklists or post-operative SSI surveillance were identified – these are promising areas for future work.

## 5 Conclusions

Although little research on how to prevent SSI in surgical practice in sub-Saharan Africa has been published, there are some encouraging signs – several high-quality studies have been undertaken in recent years and promising new methodologies and technologies are apparent. This review highlights the inconsistency of SSI definitions and follow-up methods that have been used in studies in sub-Saharan Africa in the past, and suggests that these could be resolved in the future by use of standard international definitions of SSI, such as those provided by the CDC. Important lessons can be drawn from the existing research – proper use of antibiotic prophylaxis in surgery can dramatically reduce the risk of SSI and alcohol-based preparations may provide a low-cost alternative to traditional surgical hand-washing and skin preparation methods.

## Ethical approval

None declared.

## Funding

AA is supported by Research Training Fellowship from the Wellcome Trust of Great Britain (grant number 085042).

## Conflicts of interest

All authors declare that they have no conflicts of interest. JM and AW are currently practicing surgeons and DK is a resident (trainee) surgeon in sub-Saharan Africa.

## Author contributions

AA designed the review methodology. All authors screened a portion of the titles and abstracts, and participated in discussion regarding inclusion and exclusion of papers. AA and DK extracted key data from the identified publications. AA wrote the manuscript and all authors reviewed and approved this prior to submission.

## Figures and Tables

**Table 1:** Antibiotic prophylaxis studies (*n* = 10).

Country, year of publication	Surgical procedure(s)	Intervention	Study design and RCT components	Study size	SSI definitions	Use of SWC	Follow-up period + methods	Results/notes
Uganda, 1996^11^	Variety of “abdominal” procedures	Antibiotic prophylaxis	RCT, randomized within procedure	850	From Karl et al.	No	14 days initially as IP, then via OP clinic	Single-dose pre-op ampicillin (±metronidazole) (intervention) was cheaper than extended post-op penicillin (standard) with similar rates of SSI
South Africa, 2001^3^	Caesarean section	Antibiotic prophylaxis	RCT, double blind, placebo-controlled	480	Own	No	6 weeks – as inpatient and at post-natal visit	No difference in SSI risk with pre-op cefoxitin (intervention) versus placebo.
Mozambique, 2003^12^	Caesarean section	Antibiotic prophylaxis	RCT, outcome assessor blinded	288	Own	No	7 days follow-up, r/v in OP clinic on d7	Single-dose pre-op gentamicin + metronidazole was much cheaper and as effective as extended post-op antibiotic regime (standard)
Côte d’Ivoire, 2003^32^	Orthopaedic procedures	Antibiotic prophylaxis	RCT, double blind	162	Own	NRC class used	1 yr follow-up, with r/v at d1, d8, d15, d30, 6 months, 1yr.	No difference in SSI risk between pre-op oxacillin and pre-op perfloxacin, but oxacillin cheaper.
Nigeria, 2006^4^	Clean paediatric surgery	Antibiotic prophylaxis	RCT, double blinded, placebo-control	278	Not described	No	Assessed on d5, d7, d10 by doctor	No benefit to use of ampiclox (intervention) over placebo (control) in preventing SSI in clean surgery, additional costs with use of antibiotics.
Ghana, 2007^13^	Caesarean section	Antibiotic prophylaxis	RCT, no blinding reported	320	Own	No	Not reported	Significantly lower risk of infection with intra-op amoxicillin/clavulanate (intervention) than with intra-op “ampicillin + gentamicin + metronidazole” (standard).
Nigeria, 2008^14^	Caesarean section (elective)	Antibiotic prophylaxis	Multicentre RCT, patients blinded	200	Own	No	7 days IP follow-up, with r/v on d3 and d5	No significant difference between single-dose intra-op ceftriaxone (intervention) versus post-op gentamicin + ampiclox + metronidazole (standard)
Nigeria, 2008^16^	Inguinal hernia	Antibiotic prophylaxis	RCT, no blinding reported	88	NRC	No	32 day follow-up with r/v on d4, d11, d32	Pre-op single-dose gentamicin (intervention) was associated with significantly less risk of wound infection than no antibiotic (control).
Tanzania, 2009^9^	Wide variety of procedures	Antibiotic prophylaxis	“Before and after” intervention	803	CDC	Yes	30 day, with travel expenses + meal paid for follow-up OP visit	Compared various post-op antibiotics (“before”) with single-dose pre-op amoxicillin/clavulanate (“after”) with 80% reduction in SSI risk for “after” arm.
Ethiopia, 2010^15^	Obstetric fistula repair	Antibiotic prophylaxis	RCT, single blinded	722	Own	No	Not clear from paper	Single-dose pre-op gentamicin (intervention) as effective as extended post-op regime of antibiotics (control).

Note: the following abbreviations are used in Tables 1–4: RCT, randomised controlled trial; IP, inpatient; OP, outpatient; r/v, review; SSI, surgical site infection; CDC, Centres for Disease Control; d5, 5th post-operative day; w4, 4th post-operative week; 3m, 3 months; 1yr, 1 year; NRC, National Research Council, USA; O + G, Obstetrics and Gynaecology; SWC, Surgical Wound Class (=Altemeier Class); and VP, ventriculo-peritoneal.

**Table 2:** Pre-operative intervention studies (*n* = 4).

Country, year of publication	Surgical procedure(s)	Intervention	Study design and RCT components	Study size	SSI definitions	Use of SWC	Follow-up period + methods	Results/notes
South Africa, 2001^33^	Caesarean section	Adhesive plastic drapes	Double blind RCT	605	Own	No	Wound assessed by clinician on post-op d2, d3, d4, d5	No evidence of any benefit from use of plastic drapes (no reduction of SSI nor reduction in admission length).
Nigeria, 2001^17^	Inguinal hernia	Skin preparation	RCT, no report of randomization method	200	Not described	No	R/v at d5–d10 (suture removal) and w4–w8	No difference in SSI risk between market soap + methylated spirit (intervention) and povidone-iodine (control), but former (presumed) cheaper
Côte d’Ivoire, 2009^8^	Various O + G proceedures	Surgical hand-wash	“Before and after” intervention	318	CDC	Yes	30 days – seen on alternate days	No difference in SSI risk between alcohol handrub (intervention) and povidone-iodine (standard). Alcohol handrub much more cost-effective.
Kenya, 2010^6^	Wide variety of procedures	Surgical hand-wash	Cluster RCT, crossover design	3317	CDC	Yes	30 days, OP clinic r/v and telephone calls for follow-up	No significant difference in SSI risk between soap + water (standard) and alcohol handrub (intervention), with similar costs.

**Table 3:** Intra-operative intervention studies (*n* = 6).

Country, year of publication	Surgical procedure(s)	Intervention	Study design and RCT components	Study size	SSI definitions	Use of SWC	Follow-up period + methods	Results/notes
Tanzania, 2000^19^	Caesarean section	Misgav-Ladach technique	RCT, no blinding reported	339	Not described	No	Inpatient period only	No difference in SSI risk between ML technique (intervention) and standard midline incision. Less blood loss, sutures and shorter op with ML technique
Kenya, 2001^5^	Caesarean section	Misgav-Ladach technique	RCT but weak randomization method	160	From Karl et al	No	6 weeks – seen on d7 (discharge) and at 6w	ML technique (intervention) had lower risk of SSI than standard midline incision. Shorter op and less analgesia with intervention.
Uganda, 2005^21^	VP shunt insertion	Comparing VP shunt systems	RCT, no blinding reported	90	Not described	No	1yr follow-up: OP review at 1w, 3m and 1yr	No difference in any outcome (inc SSI) between 2 types of VP shunt, but one shunt system much cheaper (US$35) than the other (US$650).
Nigeria, 2006^18^	Caesarean section	Peritoneal non-closure	RCT, blinding not explicitly stated	54	Not described	No	Not described	No significant difference found between peritoneal closure (standard) and non-closure (intervention), but non-closure cheaper and shorter surgery duration.
South Africa, 2009^22^	Circumcision	Tara-KLamp technique	RCT, no blinding used	69	Own	No	Wound examined by clinician on d3 and 6w. Self-report at 2w.	High rate of refusal of TK technique. More adverse events with TK technique (intervention) including wound infection.
South Africa, 2009^20^	Caesarean section	Peritoneal non-closure	Observational – surgeons choice of 3 methods	692	Not described	No	10 days post-partum	Compared double, single and non-closure of peritoneum. No significant difference in risk of SSI between method, but faster surgery with non-closure

**Table 4:** Post-operative intervention studies (*n* = 4).

Country, year of publication	Surgical procedure(s)	Intervent	Study design and RCT components	Study size	SSI definitions	Use of SWC	Follow-up period + methods	Results/notes
South Africa, 2000^23^	Caesarean section (emergency)	Wound drainage	RCT, no blinding used	440	From Wells et al	No	Assessed daily while IP until discharge/up to d7	No difference in SSI risk or admission length between use of drain (intervention) and no drain (standard).
Nigeria, 2000^34^	Caesarean section	Early discharge	RCT, outcome assessor blinded	100	Not described	No	Wound examined on d3 and d7 only.	No difference in SSI risk with early discharge and marked psychological benefit of early discharge.
Nigeria, 2008^24^	Mastectomy	Wound drainage	RCT, no blinding used	50	Not described	No	At least 1 month via OP clinic	No difference in wound infection risk or other outcomes between suction drain and simple drain, but simple drain much cheaper.
Nigeria, 2010^25^	Thyroid surgery	Wound drainage	RCT, no blinding reported	67	Not clearly described	No	Not described	Higher incidence of wound infection with use of drains, resulting in increase inpatient stay and costs.
